# Design of Aerogels, Cryogels and Xerogels of Alginate: Effect of Molecular Weight, Gelation Conditions and Drying Method on Particles’ Micromeritics

**DOI:** 10.3390/molecules24061049

**Published:** 2019-03-17

**Authors:** Rosalía Rodríguez-Dorado, Clara López-Iglesias, Carlos A. García-González, Giulia Auriemma, Rita P. Aquino, Pasquale Del Gaudio

**Affiliations:** 1 Department of Pharmacy, University of Salerno, 84084 Fisciano, Italy; rosalia_dorado@hotmail.com (R.R.-D.); gauriemma@unisa.it (G.A.); aquinorp@unisa.it (R.P.A.); 2 Drug Discovery and Development, University of Salerno, 84084 Fisciano, Italy; 3 Department of Pharmacology, Pharmacy and Pharmaceutical Technology, R+D Pharma group (GI-1645), Faculty of Pharmacy and Health Research Institute of Santiago de Compostela (IDIS), Universidade de Santiago de Compostela, E-15782 Santiago de Compostela, Spain; clara.lopez.iglesias@rai.usc.es

**Keywords:** aerogel, alginate, prilling, supercritical-CO_2_, cryogel

## Abstract

Processing and shaping of dried gels are of interest in several fields like alginate aerogel beads used as highly porous and nanostructured particles in biomedical applications. The physicochemical properties of the alginate source, the solvent used in the gelation solution and the gel drying method are key parameters influencing the characteristics of the resulting dried gels. In this work, dried gel beads in the form of xerogels, cryogels or aerogels were prepared from alginates of different molecular weights (120 and 180 kDa) and concentrations (1.25, 1.50, 2.0 and 2.25% (*w*/*v*)) using different gelation conditions (aqueous and ethanolic CaCl_2_ solutions) and drying methods (supercritical drying, freeze-drying and oven drying) to obtain particles with a broad range of physicochemical and textural properties. The stability of physicochemical properties of alginate aerogels under storage conditions of 25 °C and 65% relative humidity (ICH-climatic zone II) during 1 and 3 months was studied. Results showed significant effects of the studied processing parameters on the resulting alginate dried gel properties. Stability studies showed small variations in aerogels weight and specific surface area after 3 months of storage, especially, in the case of aerogels produced with medium molecular weight alginate.

## 1. Introduction

Aerogels are nanostructured materials with up to virtually 100% overall porosity in the mesoporous range and with full pore interconnectivity. Aerogels are being designed in a myriad of morphologies (e.g., cylinders, beads, microparticles) and configurations (e.g., core-shell, coated particles) conferring them with an attractive processing versatility [1,2,3]. These properties of aerogels have been exploited, particularly for silica aerogels, in the development of thermal insulation systems and Cherenkov detectors for construction and aerospace industries. The advent of novel aerogel sources from natural polymers (e.g., polysaccharides, proteins) and the development of hybrid aerogels have extended the possibilities of these materials for new applications and fields like biomedical and environmental applications [2,4,5,6]. Gel source (chemical structure, molecular weight, concentration) and chemical modifications of gels (co-gelation, post-gelation) strongly influence the textural properties of the resulting aerogels as well as the choice of the drying method. In general, supercritical drying of gels is the only process able to remove the solvent of the gel without damaging the gel network structure due to the intrinsic absence of surface tension in the pores of the gel [7,8,9,10,11]. Atmospheric drying of gels usually leads to the collapse of the pores due to the high capillary pressure gradients obtained upon the solvent removal, whereas the freeze drying of hydrogels often leads to cracks and the formation of large pores due to the increase in volume of water (i.e., the gel solvent) upon crystallization [12,13]. Xerogels and cryogels are obtained after atmospheric drying and freeze drying of gels, respectively. However, there are certain cases where aerogels can be obtained when using the latter techniques: the crosslinking of silica with polymers renders hybrid gels with enhanced mechanical strength able to lead aerogels by atmospheric drying without significant pore collapse [14]. In certain cases, freeze drying can avoid the macroporous 2D-sheet morphology of cellulose cryogels by increasing the cooling rate of the hydrogel precursors with e.g., liquid propane, or by using the spraying freeze drying approach resulting in aerogels with moderate textural properties (BET-specific surface areas of 70–100 m^2^/g) [15]. Interestingly, the freeze drying of alcogels from resorcinol-formaldehyde using *t*-butanol as solvent resulted in a significant improvement of the mesoporosity of the resulting aerogels if compared to the freeze drying of hydrogel counterparts [16]. In the case of the supercritical drying of gels, the effects of an overestimation of the processing time on the end textural properties can be negligible or noticeable depending on the gel source [4,17,18]. Overall, the choice of the drying method and the optimization of the drying process from the materials performance and economy of the process points of view will depend on each specific case of gel source and gelation mechanism.

Alginate gels in the wet and dry forms are of relevance in several research fields like thermal insulation, wound healing, drug delivery, tissue engineering, absorption or catalysis [19,20,21,22,23]. The study of the physicochemical properties of these alginate-based materials as a function of the processing variables (alginate concentration, gelation conditions, drying method) is of utmost importance. Alginate is a linear polysaccharide formed by β-d-mannuronic acid (M) and α-l-guluronic acid (G) that is obtained from brown algae or from certain bacterial strains (e.g., *Pseudomonas aeruginosa*) [24]. The alginate source determines the G-to-M ratio and the molecular weight of the polysaccharide leading to differences in the physicochemical and mechanical properties of the resulting gels [22,25]. Alginate gels and the subsequent aerogels have been obtained by ionic gelation, acidic gelation or by gelation in a primary alcohol [25,26]. Ionic gelation of alginate with divalent cations, typically Ca^2+^, usually follows the “egg-box” model with gel structures dependent on the gelation kinetics that can be tuned with the controlled release of the cation, the temperature and the cation nature [27,28,29,30]. In the acidic gelation, the intrinsic acidity of alginic acid or the addition of an acid to the sodium alginate salts results in gel formation by hydrogen bonding bridges between carboxylic groups from different polysaccharide chains [31,32,33]. The gelation of alginate in alcoholic baths takes place due to an increase of the hydrophobic interactions between the polysaccharide chains that is dependent on the polymer type [34]. Textural properties of the alginate aerogels obtained after supercritical drying of the gels resulted in BET-specific surfaces areas ranging from less than 5 m^2^/g to 608 m^2^/g and with BJH-specific pore volumes of up to ca. 7 cm^3^g^−1^ depending on the gelation approach and drying method used [27,35,36,37,38].

The analysis of the state-of-the-art unveils a paucity of information on the effect of the alginate physicochemical properties (molecular weight, M-G ratio) and gelation conditions on the textural properties of the resulting aerogels. The optimization of the processing variables for the supercritical drying of alginate aerogel beads was previously reported [39]. However, to the best of our knowledge the effect of the drying method on the textural properties of alginate gels has not been studied yet. Moreover, there is an absence of knowledge for aerogels regarding their stability upon storage. This effect is of utmost importance for drug delivery where drug products can undergo physical, chemical and biopharmaceutical degradation under storage and may result in reduced therapeutic effect and toxicity from degradation products among others. Therefore, stability tests should be taken into account in the development of a drug product and are required by regulatory agencies for approval. For these purposes, the International Council for Harmonisation (ICH) has set a classification of five climatic zones for stability evaluation [40,41].

In this work, alginate gel beads of narrow size distribution were obtained by the prilling technique [42,43] followed by their drying by supercritical drying, freeze-drying and oven-drying. A systematic study of the alginate aerogels, cryogels and xerogels performance was conducted to get insights on the effect of alginate molecular weight, alginate solution concentration, gelation solution (ionic gelation in aqueous or ethanolic CaCl_2_ solution) and drying method (oven drying, freeze-drying, supercritical drying). Textural properties, morphology and densities of the processed samples were studied and the effects of the processing variables and storage conditions of ICH climatic zone II identified, with the purpose to get adequate porous materials with proper surface areas and porosities that could be useful as carriers for topical administration as, for example, in wound healing.

## 2. Results and Discussion

### 2.1. Effect of Alginate Molecular Weight, Alginate Concentration and Gelling Solution Properties on Aerogel Beads

Alginate beads were produced from alginates of medium (MMW) and high (HMW) molecular weight as well as using different solution concentrations (1.25–2.25% (*w*/*v*)) to evaluate their influence on the physicochemical and textural properties of the resulting alginate aerogel beads. Moreover, both hydrogel and alcogel alginate particles were obtained by gelation of alginate solution droplets in either aqueous or ethanolic CaCl_2_ 0.3 M solution.

Diameter of alginate beads obtained by prilling is the result of various variables concurring in producing a gelled matrix from alginate droplets. In a previous work [44] extensive study of the process parameters in term of nozzle diameter, feed rate of the solution allowed proper setting of the production of droplets with desired size. However, small changes in setting the proper operating variables allowed the production of beads with narrow size distribution with a relative standard deviation lower than 5% (see Table 1).

Hydrogel bead diameters (d = 2.2–2.6 mm) were mainly larger than those of alcogels (d = 2.3–2.4 mm) with differences in beads diameter behaviour depending on alginate molecular weight. This difference in size was reduced with an increasing concentration of the alginate solution when MMW alginate was used, whereas a reverse trend was obtained when HMW alginate was used (Table 1). This behaviour can be explained taking into account the different formation of association structures through interpolymer bridging in aqueous or ethanolic gelling bath that leads to variations in viscosity of the gelling droplet and consequently to a variation in its gelation rate [45]. Such phenomenon also influenced the particle size of aerogels from with a slightly lower diameter for high molecular alginate (HMW) than for MMW aerogels. Moreover, HMW alginate gel beads experimented a higher shrinkage after the supercritical drying process, thus, they also resulted to be less porous than aerogels obtained with MMW alginate. Alcogels MMW125 and MMW150 showed a swelling behaviour instead of shrinkage after the scCO_2_ drying process (Table 1). This could be due to the immediate contraction in volume of the polymer matrix in the CaCl_2_ ethanolic solution during the prilling step, thus reducing the subsequent shrinkage during the supercritical drying [46].

**Figure 1:**
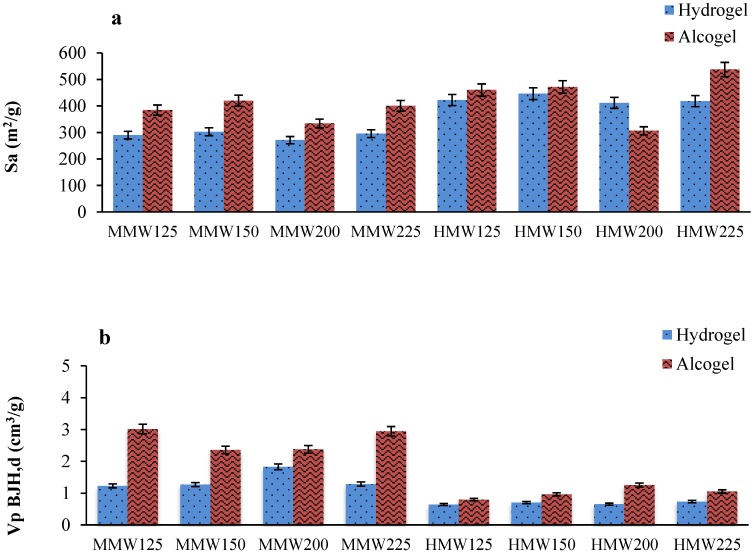

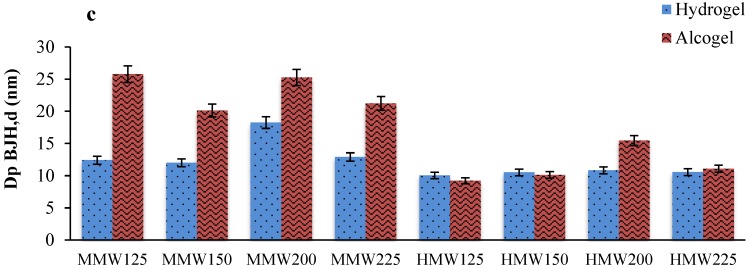
Textural characterization of alginate aerogels from different alginate sources, aqueous concentrations and gelation conditions (aqueous-in blue- or ethanolic-in red- solutions); (**a**) Specific surface area (m^2^/g), S_a_; (**b**) BJH cumulative desorption pore volume (cm^3^/g); (**c**) BJH-pore diameter of aerogels.

The specific surface areas (S_a_) of aerogels obtained by supercritical drying were in the range 271.0–537.3 m^2^/g (Figure 1a). Aerogels obtained from gel beads formed in ethanolic CaCl_2_ solutions showed higher S_a_ values than aerogels derived from gel beads formed in aqueous CaCl_2_ solutions. The ethanolic gelling bath promoted the shrinkage of the polymeric matrix structure during the gel beads formation lead to smaller particles and subsequently to aerogel with higher specific surface area, with the exception of the HMW200 sample. S_a_ value was also depending on alginate molecular weight used to produce beads: higher S_a_ values were obtained in the case of aerogels produced with HMW alginate (306.2–537.3 m^2^/g) than those produced with MMW alginate (271.0–419.6 m^2^/g). Regarding the pore size distribution, aerogel beads derived from alcogels presented higher BJH- pore volumes (Vp_BJH,d_ = 0.80–3.02 cm^3^/g) than the aerogels derived from hydrogels (0.64–1.83 cm^3^/g) (Figure 1b). Differences in gelation rate due to the presence of more carboxylic groups on a single polymeric chain was also responsible of the Vp_BJH,d_ variations between aerogels produced from MMW or HMW alginate source. For HMW alginate aerogels, there is a smaller amount of internal gaps due to a major packing between the polymer chains. A similar trend was also observed for the pore diameter (Dp_BJH,d_), with the exception of HMW125 and HMW150 aerogels where no significant differences between aerogels from hydrogels and alcogels were obtained. Pore diameters in the mesoporous range were obtained for all aerogels (9.2–25.8 nm) (Figure 1c).

**Figure 2:**
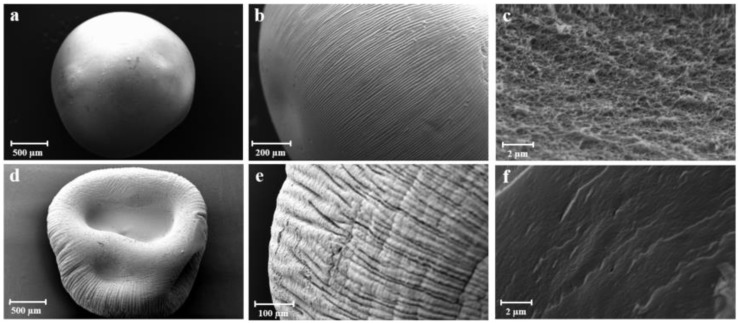
SEM images of dried HMW225 alcogel: spherical beads (**a**), magnification on the smooth surface (**b**), inner porous network (**c**) of aerogels; collapsed structure (**d**), magnification of the surface (**e**) and inner structure (**f**) of cryogels.

Hydrogel beads were also freeze-dried obtaining cryogels that showed lower specific surface areas (0.8–245.6 m^2^/g) than its aerogel counterparts, while textural properties of the xerogels (oven-dried gels during ≥ 48 h) could not be studied since their porosity was below the detection limit of the N_2_ adsorption-desorption equipment. As expected, cryogels and xerogels showed lower textural properties than their aerogel counterparts. In fact, SEM analyses demonstrated the sphericity of the obtained aerogel beads (Figure 2a), the presence of the characteristic smooth surface of alginate aerogels (Figure 2b) as well as the preservation of the porous nanostructure of the matrix (Figure 2c). On the contrary, cryogel beads obtained from alcogels exhibited partial collapsed structure (Figure 2d) and reduced porosity (Figure 2f). In addition, it was also observed an intense white colour for the alginate beads treated with supercritical drying, indicating a complete purification of the original alginate [30,39] whereas the cryogel beads showed pale yellow colour (Figure S1).

### 2.2. Aerogel Beads Stability

The stability under storage of the aerogels was tested after 1 and 3 months under the humidity and temperature conditions of ICH-climatic zone II. These conditions are usually taken for stability tests of drug products to cover the climatic zones I and II corresponding to most of the territory of Europe, USA and Japan [40].

Differences in stability between aerogels obtained from hydrogels and alcogels were evaluated using samples produced at the same concentration and alginate molecular weight (HMW150). Moreover, aerogels obtained from alcogels at the same polymer concentration (2.25% (*w*/*v*)) were used to assess the influence of alginate molecular weight on aerogel stability. After 1 month of storage, aerogels did not show significant differences in weight, whereas an increase in weight between the 3.6% and 32.5% was observed after 3 months, probably due to the uptake of humidity. The gain in weight upon a 3-month storage of HMW150 aerogels obtained from the hydrogel and the alcogel showed an increase of 3.6% and 13.1%, respectively. This phenomenon can be explained by the fact that starting aerogels (*t* = 0) obtained from alcogels have higher specific surface areas and pore volumes than those obtained from hydrogels, thus conferring them a higher ability to uptake humidity and, as a consequence, their weight increase easier. The gain in weight for 2.25% (*w*/*v*) alginate concentration aerogels was higher for alginates of higher molecular weight (32.5% in the case of HMW225 and 21.2% in the case of MMW225). These differences can be explained considering that the starting aerogels obtained from HMW alginate have higher specific surface areas and pore volumes than those ones obtained from MMW alginate due to the presence of more carboxylic groups for a single chain of alginate able to interact with the Ca^2+^ ions during the prilling production of the gel beads.

**Figure 3:**
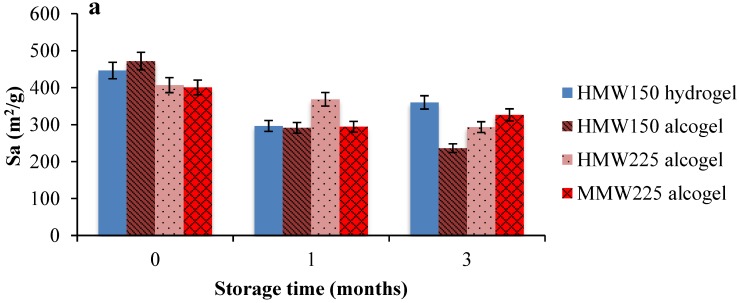

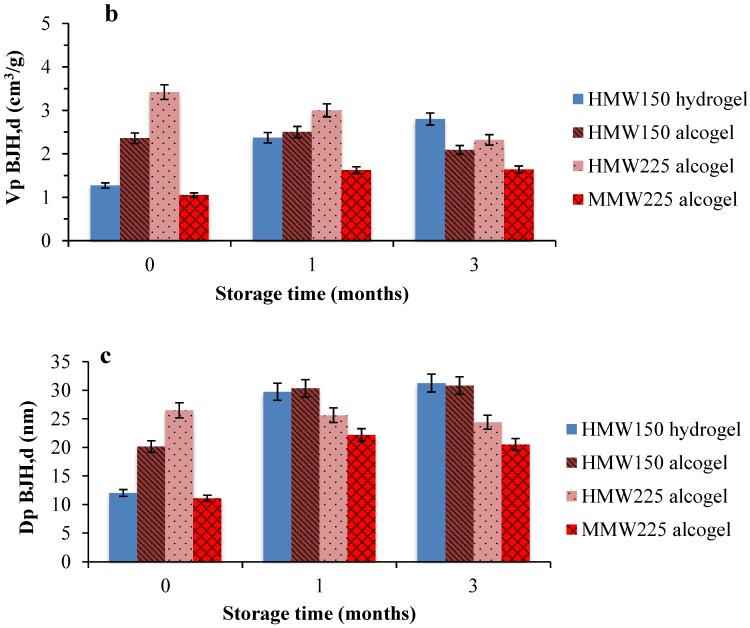
Effect of stability tests under storage (25 °C, 65% r.h.) during three months on textural properties of alginate aerogels; (**a**) Specific surface area (m^2^/g), S_a_; (**b**) BJH cumulative desorption pore volume; (**c**) BJH-mean pore diameter. Mean values ± SD.

Specific surface area (S_a_) decreased in all cases after 3 months of storage probably due to the increasing in pore size related to the uptake of humidity. For HMW150 aerogels, the reduction was higher in aerogels produced from alcogels (ca. 50%) due to the fact that the originating alcogels demonstrated higher surface areas than hydrogels, leading to higher adsorption capacity being able to uptake higher amounts of humidity. The effect of alginate molecular weight on the Sa values was less pronounced after 3-months storage with variations of 18.5% and 28.0% for MMW225 and HMW225, respectively (Figure 3). BJH pore volume and pore diameter of the beads demonstrated similar behaviour during the storage conditions showing main variations after 1-month storage. Aerogels obtained by hydrogels with the lowest concentration in alginate demonstrated the highest increase, whereas its homologous obtained by alcogels showed only slight variations in BJH pore volume (about 10%) while pore diameter increased of about 45% after one month then it remained almost constant over time, as shown in Figure 3b,c.

## 3. Materials and Methods

### 3.1. Materials

High viscosity sodium alginate from brown algae (HMW, 180 kDa, 1% viscosity: 65 mPa·s; M/G ratio 70/30) was purchased from Carlo Erba (Milan, Italy) and medium viscosity sodium alginate from brown algae (MMW, 120 kDa, 1% viscosity: 40 mPa·s; M/G ratio 70/30) from Dompè Pharma (L’Aquila, Italy). Calcium chloride (CaCl_2_, 93% purity) was obtained from Sigma-Aldrich (Milan, Italy) and absolute ethanol (≥99.8% purity) from VWR (Llinars del Vallés, Spain). Carbon dioxide (99.8% purity) from Praxair (Madrid, Spain) was used for the supercritical drying of the gels. All other reagents were purchased from Sigma-Aldrich (Milan, Italy) and employed as received.

### 3.2. Methods

#### 3.2.1. Alginate Gel Beads Preparation

Stock aqueous alginate solutions were prepared at different concentrations (1.25, 1.50, 2.00 and 2.25% (*w*/*v*)). Batches from different molecular weight alginates (HMW and MMW) were used. Gel beads were prepared using a prilling apparatus (Büchi Encapsulator, Büchi Labortechnik AG, St. Gallen, Switzerland) equipped with a 400 µm-diameter nozzle. Alginate solutions (60 mL) were pumped at a flow rate of 8 mL/min. Nozzle vibration was set at a frequency of 350 Hz and an amplitude of 100%. CaCl_2_ 0.3 M in either aqueous or ethanolic solution was used as gelling agent. The distance between the nozzle and the gelling bath was set at 8 cm. After the droplets fall into the bath, the hydrogel beads formed were stirred (ageing time) for 5 min considering zero time the end of the prilling process, whereas the alcogel beads where immediately collected after the end of the prilling process; thereafter, they were rinsed with either distilled water or ethanol depending on the type of solution used as gelling bath, leading to the production of hydrogels and alcogels, respectively. MMW*x* samples denote those prepared from medium molecular weight alginate and HMW*x* those prepared from high molecular weight alginate, being *x* the concentration of alginate in the 1.25–2.25% (*w*/*v*) range.

#### 3.2.2. Drying of the Gel Particles

Before the drying of all the produced batches, a direct solvent exchange process to replace water or ethanol with absolute ethanol was conducted twice. Once the beads were conditioned, different drying methods were carried out, namely drying in oven at 60 °C until constant weight was reached, freeze-drying, and drying with a continuous flow of supercritical CO_2_ (scCO_2_).

Freeze-drying was carried out at ca. −80 °C and 0.01 mbar in a Telstar Lyo Quest Plus −85 °C/ECO apparatus (Barcelona, Spain) over 24 h. Hydrogel beads were previously cooled to −20 °C and followed by further chilling with liquid nitrogen (−196 °C).

Supercritical drying was carried out in a 300 mL stainless steel autoclave (Eurotechnica GmbH, Bartgteheide, Germany). The drying process was carried out using scCO_2_ at 40 °C and 120 bar during 3.5 h using a flow rate of 7 g/min during the first hour and of 5 g/min during the rest of the process. Drying experiments were carried out in triplicate. These operating conditions were chosen in accordance to the vapor-liquid equilibrium data for the ethanol-CO_2_ binary system in order to operate at conditions above the mixture critical point. Under these conditions an adequate extraction of the organic solvent of the gel is obtained whilst preserving the internal gel structure [36].

#### 3.2.3. Aerogels Characterization

##### Shrinkage of Gel Beads After Supercritical Drying

For alginate aerogels, the percentage of volume shrinkage from native gel beads to aerogels was calculated using Equation (1):

Volume shrinkage (%) = (1 − (V_aerogel beads_/V_native gel beads_)) × 100 (1)

where the average volumes (cm^3^) of the gels and aerogels were calculated by image analysis (ImageJ 1.50 software, Bethesda, MD, USA). The particle diameter of gel and aerogel beads (d) was also calculated by image analysis. For all measurements, only particles with a sphericity coefficient higher than 0.7 (>90% of total particles of each batch) were considered and at least 20 particles for each sample were measured.

##### Textural Properties of Aerogels and Cryogels

The textural properties of aerogels, cryogels and xerogels were studied by nitrogen adsorption-desorption analysis in an ASAP 2000 apparatus (Micromeritics, Norcross, GA, USA). Specific surface area (S_a_) and pore size distribution were calculated by the Brunauer-Emmett-Teller (BET) and Barret-Joyner-Halenda (BJH) methods, respectively. The overall specific pore volume (V_p_BJH,d) and the mean pore diameter (D_p_BJH,d) were also obtained by the BJH method. Samples were previously outgassed (<10^−5^ mbar) at 60 °C for at least 20 h before the analysis.

##### Density and Porosity of Aerogels

The skeletal density (ρ_skeletal_) of aerogel beads (Table S1) was measured by helium pycnometry (Micropycnometer Quanta-Chrome MPY-2, Boynton Beach, FL, USA) at room temperature and using helium as the displacement gas due to its size and inert behaviour. The envelope density (ρ_envelope_) of the alginate aerogel beads was calculated from the Equation (2):

(2) $$\rho_{\mathrm{envelope}}=\frac{m}{V\times N}$$

being *m* the sample weight, *V* the average volume of an aerogel bead, and *N* the number of beads for each sample. Overall porosity (ε) of aerogel beads was obtained from the bulk and skeletal densities (Equation (3)):

(3) $$\varepsilon =(1-\frac{\rho_{\mathrm{envelope}}}{\rho_{\mathrm{skeletal}}})\times 100$$

##### Morphological Analyses

Alginate aerogel beads were characterized in terms of external and inner porous structure by scanning electron microscopy (SEM, Zeiss Ultra Plus microscope, Zeiss, Oberkochen, Germany). Aerogels were iridium-sputtered under vacuum during ca. 10 min prior to imaging to minimize charging and to improve the image quality.

#### 3.2.4. Aerogels Stability Tests Under Storage

A known amount of alginate aerogel samples (ca. 50 mg) were placed on top of a platform inside sterile and hermetic glass vessels containing a sulphuric acid solution (37% (*v*/*v*)) at the bottom and placed in an oven at 25 °C to maintain the required relative humidity (65%) and mimic the ICH climatic conditions of zone II (Mediterranean/subtropical zone) for stability studies of drug formulations [40]. The vessels were stored under these conditions during 1 and 3 months. Once the storage period was over, the aerogels were collected for further characterization.

## 4. Conclusions

In this work, alginates with different molecular weight were used for the production of gel beads through the prilling technique in single nozzle configuration, using both aqueous and ethanolic calcium chloride solutions as gelling agent obtaining hydrogel and alcogel beads in a very narrow size distribution (ca. 2.4 mm ± 6.0% for each formulation). Supercritical CO_2_ drying of these gels allowed to obtain aerogel particles with spherical shape. Gelation in ethanolic media promoted the formation of aerogels with superior textural properties in comparison with the aerogels derived from particle gelified in aqueous media. The textural properties of aerogels were far higher than those obtained from cryogels and xerogels obtained by freeze-drying and oven drying, respectively. The alginate molecular weight had an important effect on the degree of shrinkage and the porosity during the production of alginate aerogels. The use of medium molecular weight alginate was the most suitable to reduce shrinkage and leading to aerogels with higher porosity. On the contrary, aerogels with higher surface areas where obtained when high molecular weight alginate was used. Alginate aerogels may reduce their textural properties upon storage at 25 °C and 65% of relative humidity depending on the formulation, being aerogels produced with medium molecular weight alginate especially stable after 3 months of storage under these conditions. In conclusion, the size and the high specific surface areas and porosities of those materials make them suitable as carriers for topical administration of drugs, since they are easily manageable and due to their textural properties. High surface area, in fact, allows fast absorption of biological fluids that could be useful to set controlled drug release hydrogels or by absorbing high quantities of exudates once applied on a wound could produce a first line wound dressing.

## Figures and Tables

**Table 1:** Average diameter (mm) of native gel beads (hydrogel and alcogel) and aerogels, volume shrinkage and porosity (in percentage) of each batch after supercritical drying. Mean ± SD; (*n* = 6).

Formulation	Diameter (mm)		Shrinkage (%)	ε (%)	Diameter (mm)		Shrinkage (%)	ε (%)
	Hydrogel	Aerogel			Alcogel	Aerogel		
MMW125	2.64 ± 0.20	2.52 ± 0.19	13.3	99.7 ± 0.0	2.31 ± 0.13	2.77 ± 0.23	−75.4 *	99.8 ± 0.0
MMW150	2.51 ± 0.15	2.20 ± 0.10	32.5	99.5 ± 0.0	2.38 ± 0.16	2.51 ± 0.16	−16.9 *	99.7 ± 0.0
MMW200	2.43 ± 0.09	2.36 ± 0.16	6.7	99.5 ± 0.0	2.44 ± 0.13	1.86 ± 0.02	60.9	97.6 ± 0.1
MMW225	2.41 ± 0.11	2.33 ± 0.14	10.8	99.3 ± 0.0	2.37 ± 0.13	1.96 ± 0.10	43.7	98.7 ± 0.0
HMW125	2.18 ± 014	1.93 ± 0.08	30.9	99.3 ± 0.0	2.46 ± 0.23	1.81 ± 0.02	60.8	99.2 ± 0.0
HMW150	2.34 ± 0.14	1.73 ± 0.02	60.3	98.4 ± 0.0	2.32 ± 0.12	1.55 ± 0.06	71.2	98.6 ± 0.0
HMW200	2.56 ± 0.13	2.03 ± 0.13	50.6	99.5 ± 0.0	2.33 ± 0.09	1.81 ± 0.03	53.7	99.5 ± 0.0
HMW225	2.40 ± 0.13	1.92 ± 0.08	49.3	99.2 ± 0.0	2.33 ± 0.10	1.90 ± 0.08	46.3	99.3 ± 0.0

(*) Negative values indicate a swelling behaviour instead of shrinkage after the sc-CO_2_ drying.

## Supplementary Materials

The following are available online.
