# Supplementary material for: Design of Aerogels, Cryogels and Xerogels of Alginate: Effect of Molecular Weight, Gelation Conditions and Drying Method on Particles’ Micromeritics
Source: Molecules. 2019 Mar 17;24(6):1049. doi: 10.3390/molecules24061049 (PMC6471992; doi:10.3390/molecules24061049)
Supplement: Supplementary file 1 [file molecules-24-01049-s001.pdf]

molecules

Article

Design of aerogels, cryogels and xerogels of alginate: Effect of molecular weight, gelation conditions and drying method on particles micromeritics

Table 1. Skeletal density (g/cm^3) ± SD of alginate aerogels.

Aerogel	ρ Skeletal (g/cm^3)	
	Hydrogel	Alcogel
MMW125	1.40 ± 0.02	1.44 ± 0.03
MMW150	1.33 ± 0.01	1.41 ± 0.03
MMW200	1.37 ± 0.03	1.46 ± 0.04
MMW225	1.38 ± 0.06	1.50 ± 0.05
HMW125	1.33 ± 0.06	1.44 ± 0.02
HMW150	1.48 ± 0.03	1.50 ± 0.03
HMW200	1.56 ± 0.02	1.52 ± 0.02
HMW225	1.52 ± 0.02	1.52 ± 0.02

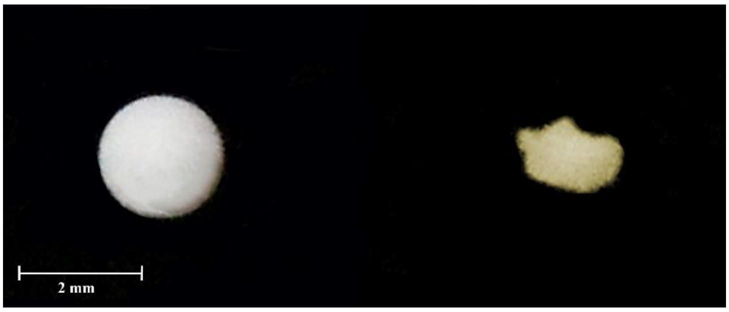

Figure 1. Aerogel (left) and cryogel (right) beads.
